# Microbial Imbalance and Stochastic Assembly Drive Gut Dysbiosis in White-Gill Diseased *Larimichthys crocea* (Richardson, 1846)

**DOI:** 10.3390/microorganisms13122737

**Published:** 2025-11-30

**Authors:** Xuan Wang, Huangwei Cheng, Ting Liu, Xuelei Wang, Xiongfei Wu, Junqi Yu, Demin Zhang, Weiliang Shen, Dandi Hou

**Affiliations:** 1 State Key Laboratory for Quality and Safety of Agro-Products, School of Marine Sciences, Ningbo University, Ningbo 315211, China; 2211130115@nbu.edu.cn (X.W.); cheng_huangwei@163.com (H.C.); liuting991122@163.com (T.L.); 13968836902@126.com (J.Y.); zhangdemin@nbu.edu.cn (D.Z.); 2 Zhejiang Key Laboratory of Aquatic Germplasm Resources, Ningbo Academy of Oceanology and Fishery, Ningbo 315832, China; xlwang126@163.com (X.W.); wxiongfei@hotmail.com (X.W.); 3 State Key Laboratory of Mariculture Breeding, Xiamen University, Xiamen 361000, China; 4 Zhejiang Key Laboratory of Coastal Biological Germplasm Resources Conservation and Utilization, Zhejiang Mariculture Research Institute, Wenzhou 325005, China; 5 Institute of One Health Science, Ningbo University, Ningbo 315211, China

**Keywords:** large yellow croaker, fish disease, intestinal homeostasis, pathobiome, *Photobacterium damselae* subsp. *damselae*, host–pathogen interactions

## Abstract

White-gill disease has emerged as one of the major health threats in large yellow croaker *Larimichthys crocea* (Richardson, 1846) aquaculture, yet its underlying microbial mechanisms remain poorly understood. In this study, we investigated the gut microbiota of healthy and white-gill diseased *L. crocea* across different growth stages and aquaculture locations using 16S rRNA gene amplicon sequencing and bioinformatics analysis. Across both juvenile and adult fish, as well as multiple sampling locations, diseased individuals consistently exhibited significantly reduced microbial richness and evenness compared to healthy counterparts, along with a clear divergence in community composition. Notably, the relative abundance of *Photobacterium damselae* subsp. *damselae* was markedly increased in diseased fish, especially juveniles, accompanied by a decline in beneficial genera such as *Bacillus*. Co-occurrence network analysis revealed simplified microbial interactions and decreased community stability in gut of diseased fish. Null model analysis further indicated that stochastic processes dominated gut microbial assembly, with a higher contribution in diseased individuals, suggesting weakened host selection pressure and enhanced random colonization under disease conditions. These findings highlight the important role of gut microbiota dysbiosis in the development of white-gill disease and provide new insights into microbiota-based diagnostics and ecological strategies for disease prevention in marine aquaculture.

## 1. Introduction

The large yellow croaker *Larimichthys crocea* (Richardson, 1846) is one of the most economically important marine fish species in China and serves as a key pillar of the national aquaculture industry [1]. According to the China Fisheries Statistical Yearbook 2024 [2], it ranks among the most productive mariculture fish species in the country, with annual yields exceeding 280,000 tons. However, the rapid intensification of aquaculture, characterized by higher stocking densities, nutritional imbalances, and environmental stressors, has led to a surge in disease outbreaks, posing serious challenges to the sustainability of the industry [3]. Among these, white-gill disease has emerged as a particularly severe threat to the health and survival of large yellow croaker, causing considerable economic losses and severely disrupting production stability [4].

White-gill disease usually occurs during the summer months when water temperatures exceed 25 °C, affecting both juvenile and adult *L. crocea*. In recent years, it has become increasingly prevalent in net-cage mariculture, particularly in the southeastern coastal provinces of Zhejiang and Fujian [5]. The disease has a rapid onset, high spread rate, and prolonged epidemic period, with a cumulative mortality rate ranging from 20% to 50% [6,7]. Affected fish generally present with intact external surfaces, pale body coloration, and distinctive white gill filaments, along with enlarged livers and kidneys [5]. Despite the widespread impact of this disease, there are currently no effective prophylactic or therapeutic measures, mainly due to the fact that its etiology remains unresolved. Some studies have suggested a viral origin, particularly iridovirus-like particles [6,7,8], while others have implicated parasitic Myxosporea [9]. It is now generally accepted that white-gill disease is a multifactorial disease, likely resulting from a combination of pathogenic infections and host metabolic disorders [4,5]. The pathogenesis of white-gill disease urgently needs innovative research ideas to break through the stagnation of current prevention and control work.

The rapid advancement of modern pathology and microbiology has challenged the traditional “one pathogen-one disease” paradigm, leading to adoption of the “pathobiome” concept across human, animal, and plant disease research [10]. This innovative concept emphasizes the complex interactions between host-associated microbiota (particularly the gut microbiota), the host itself, and environmental factors in disease etiology [11], providing a powerful theoretical framework for deciphering multifactorial diseases like white-gill disease.

The gut microbiota plays a critical role in the overall health of fish, functioning as a key metabolic and immune organ that influences digestion, nutrient absorption, and pathogen defense [12]. A balanced and diverse microbial community is essential for maintaining intestinal homeostasis. Disruption of this balance, known as gut microbiota dysbiosis, can compromise immune function, increase susceptibility to opportunistic infections, and contribute to disease onset [13]. Substantial evidence from various fish species, including largemouth bronze gudgeon (*Coreius guichenoti*) with furunculosis [14], crucian carp (*Carassius auratus*) with “red-operculum” disease [15], and zebrafish infected with *Streptococcus agalactiae* [16], demonstrates consistent patterns of decreased gut microbial diversity and increased pathogenic loads during disease outbreaks. These findings underscore gut microbiota dysbiosis as a key factor in fish disease pathogenesis, with emerging research revealing its broader influence on disease progression through immune modulation and metabolic regulation [17,18].

Despite increasing reports of white-gill disease in *L. crocea* and its associated severe economic impact, the underlying mechanisms of this disease remain poorly understood. Previous studies have primarily focused on identifying potential pathogens or describing external pathological features, yet the role of the gut microbiota in the onset and progression of white-gill disease has received limited attention. Given that many aquatic diseases are increasingly recognized as microbiome-mediated disorders rather than infections caused by single pathogens [10], this pathogen-centered perspective may be insufficient to explain the complex, recurrent, and multifactorial nature of white-gill disease. However, a comprehensive ecological understanding of gut microbiome during the pathogenesis of this disease is still lacking. In particular, how microbial co-occurrence networks and the ecological processes governing community assembly are altered under disease conditions remains largely unexplored. This critical knowledge gap hampers a holistic understanding of disease etiology and limits the development of effective microecology-based prevention and control strategies.

To address this gap, the present study conducted systematic sampling during white-gill disease outbreaks across multiple net cages in Ningbo, Zhejiang Province, a representative major production area for *L. crocea* aquaculture in China. Using 16S rRNA gene amplicon sequencing combined with comprehensive bioinformatics analysis, we compared the gut microbiota of healthy and white-gill diseased fish in terms of community diversity, taxonomic composition, co-occurrence networks, and microbial assembly processes. This study aims to provide a deeper insight into the ecological role of the gut microbiota in white-gill disease, thereby establishing a scientific foundation for the development of microbiota-based diagnostic and preventive strategies.

## 2. Materials and Methods

### 2.1. Sample Collection

**Figure 1:**
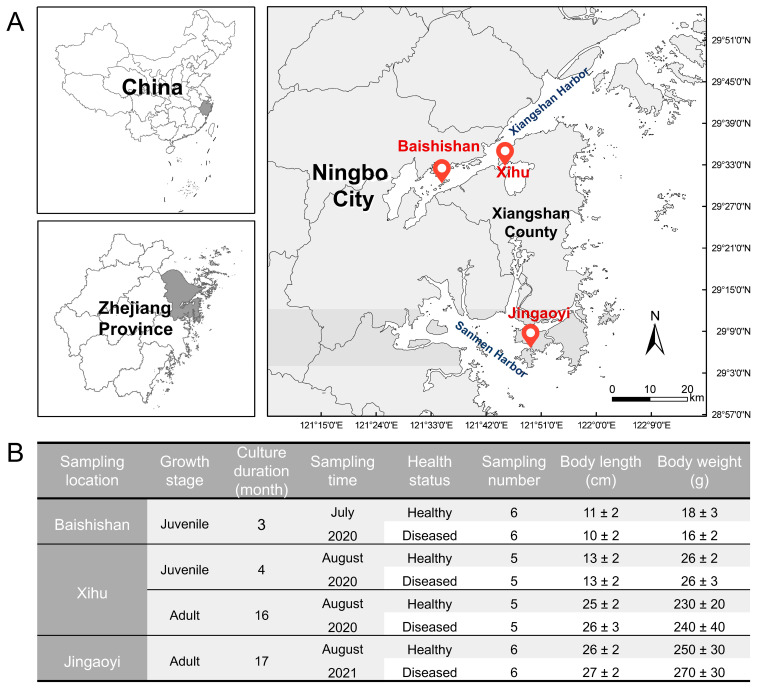
Sampling sites and collections. (**A**) Map of sampling locations. (**B**) Description of samples collected for this study.

Samples of healthy and diseased *L. crocea* (Daiqu) were collected from cages in three maricultural sites around Xiangshan County (Ningbo City, Zhejiang Province, China) during outbreaks of white-gill disease in the summer (July–August) of 2020 and 2021. The three maricultural sites are located in Baishishan Island (briefly named Baishishan, 121°36′3″ E, 29°30′22″ N), Xihu Harbor (briefly named Xihu, 121°46′9″ E, 29°32′48″ N) and Jingaoyi Harbor (briefly named Jingaoyi, 121°50′6″ E, 29°6′11″ N; Figure 1A). Sample information and the environmental parameters of each sampling site are detailed in Figure 1B and Table S1, respectively. The sample size used in this study (*n* = 5–6 per group) was determined based on the availability of fish exhibiting typical, non-confounding white-gill symptoms and represents the minimum required to achieve statistical robustness while adhering to the 3Rs principles. The strong consistency and significant differences observed across individuals confirm that this sample size was adequate to detect the core microbial shifts associated with the disease.

The fish were anesthetized with eugenol (100 mg L^−1^; Adamas, Genève, Switzerland). After the body surface was sterilized with alcohol (75%), each fish was dissected with sterilized scissors and tweezers. A standardized midgut segment (~2–3 cm) was aseptically collected and immediately transferred to sterilized centrifuge tubes (2 mL) and quickly dropped into liquid nitrogen. Samples of the culture environments, including feed, seawater, and cage attachment (particulate matter, biofilm, etc., which were attached to the cage-seawater interface and could be ingested by *L. crocea* during mariculture), were also collected. Seawater samples were pre-filtered with 100 μm sterilized nylon mesh and then filtered through a 0.22 μm polycarbonate membrane (Millipore, Burlington, MA, USA). All samples were stored in liquid nitrogen and then at −80 °C after being transported back to the laboratory until DNA extraction.

### 2.2. Histopathological Examination

All collected fish were dissected on site, and the clinical symptoms on the body surface and in body were observed and photographed. In order to further explore the pathological characteristics of white-gill disease, three healthy and three diseased fish were randomly selected for histopathological observation. Gill and liver tissues were collected and fixed in 4% paraformaldehyde. The fixed tissues were dehydrated with gradient ethanol and embedded in paraffin wax. Serial sections (5 μm thickness) were cut from each paraffin block using a microtome, and 3–4 sections were collected. One representative section per block was mounted on slides, dried, and stained with hematoxylin and eosin (H&E) following standard protocols. Finally, the stained slides were observed under a microscope (Nikon Eclipse Ci, Tokyo, Japan) and scanned with an imaging system (Nikon DS_U3, Tokyo, Japan).

### 2.3. DNA Extraction, 16S rRNA Gene Amplification and Illumina Sequencing

A total of 44 gut samples (healthy: *n* = 22; diseased: *n* = 22), as well as 13 seawater, 9 feed, and 13 cage-attachment samples, were used for 16S rRNA gene sequencing. The total microbial genomic DNA from gut, feed and cage-attachment samples was extracted using QIAamp^®^ DNA Stool Mini Kit (Qiagen, Hilden, Germany), while the seawater microbial genomic DNA was extracted using DNeasy^®^ PowerSoil Kit (Qiagen, Hilden, Germany). The V4 region of 16S rRNA gene was amplified using dual-indexed bacterial universal primers 515F (5′-GTGCCAGCMGCCGCGGTAA-3′) and 806R (5′-GGACTACHVGGGTWTCTAAT-3′) [19]. An aliquot of 10 ng of purified DNA template from each sample was amplified in 15 μL PCR reaction system. The amplification conditions were as follows: initial denaturation at 98 °C for 1 min, followed by 30 cycles of denaturation at 98 °C for 10 s, annealing at 50 °C for 30 s, and extension at 72 °C for 30 s, with a final extension at 72 °C for 5 min. During DNA extraction and PCR amplification, negative controls (using DNA-free water as template) were included to detect any potential contamination. No detectable amplification was observed in these controls. The amplification products were purified with Qiagen Gel Extraction Kit (Qiagen, Hilden, Germany). Sequencing libraries were generated using TruSeq^®^ DNA PCR-Free Sample Preparation Kit (Illumina, San Diego, CA, USA). The library quality was evaluated using the Qubit @ 2.0 Fluorometer (Thermo, Waltham, MA, USA) and Agilent BioAnalyzer 2100 (Agilent, Santa Clara, CA, USA) systems. Paired-end (2 × 250 bp) sequencing was performed using the Illumina NovaSeq platform (Illumina, USA).

### 2.4. Sequence Processing

The raw sequences were demultiplexed with QIIME v1.9.1 [20]. The sequences were quality filtered at a Phred quality score (Q) < 20 using the script split_libraries_fastq.py. The chimeras were identified and removed by USEARCH v11.0.667 [21]. Sequences with a similarity higher than 99% were clustered into the same operational taxonomic unit (OTU) using the script pick_open_reference_otus.py. A 99% similarity threshold was applied to increase taxonomic resolution for closely related gut-associated taxa, particularly opportunistic pathogens, as higher thresholds have been shown to improve discrimination of near-identical 16S rRNA gene sequences compared with the traditional 97% cutoff [22,23], while remaining a practical choice when Amplicon Sequence Variant (ASV)-level inference is not applied. The representative sequences were taxonomically assigned against the SILVA 132 database. Archaea, chloroplasts, mitochondria, and the sequences that were not assigned to bacteria were removed. To normalize the sequencing depth of each sample, the OTUs table was rarefied at 7300 sequences per sample, based on the minimum sequencing depth. Rarefaction curves were generated to evaluate sequencing completeness, all of which approached a plateau, indicating sufficient coverage of microbial diversity (Figure S1).

### 2.5. Statistical Analysis

Alpha diversity (Observed species, Shannon index and Pielou’s evenness) and Bray–Curtis distance of microbiota were calculated by the R package “vegan” (v2.5-3) [24]. The assumptions of normality and homogeneity of variances for α-diversity data were verified using the Shapiro–Wilk and Bartlett’s tests, respectively (*p* > 0.05 for all tests). Subsequently, one-way analysis of variance (ANOVA) followed by Tukey’s Honestly Significant Difference (Tukey’s HSD) post hoc test was applied to evaluate significant differences among groups. Principal coordinate analysis (PCoA) based on Bray–Curtis dissimilarity characterized the compositional differences in bacterial communities, and analysis of similarities (ANOSIM) was used to test the significance of compositional differences among different groups. Permutational multivariate analysis of variance (PERMANOVA) with 999 permutations was performed to characterize the influence degree of various factors on the composition of gut bacterial community of *L. crocea*. Similarity percentage analysis (SIMPER) in the R package “vegan” was applied to identify key OTUs primarily responsible for the community dissimilarity between healthy and diseased *L. crocea*. For co-occurrence network construction, the top 200 OTUs were selected based on their highest relative abundances across all samples to minimize noise and focus on ecologically relevant taxa [25,26]. Pairwise correlations among OTUs were calculated using Spearman’s rank correlation by the R package “Hmisc” (v4.0-6) [27]. Only strong and statistically significant correlations were retained (|*r*| > 0.6 and *p* < 0.05). The co-occurrence network was constructed using the R package “igraph” (v2.1.4) [28], while network visualization and topological parameter calculations were performed using Gephi (v0.10.1) [29]. Null modelling approach was used to quantify the relative influence of the processes governing gut microbiota assembly in *L. crocea* [30]. Phylogenetic turnover between communities was estimated by abundance-weighted β-mean nearest taxon distance (βMNTD) metric calculated by the R package “picante” (v1.8.3) [31]. The distribution of null βMNTD (βMNTD_null_) was created by randomly shuffling OTUs on the phylogenetic tree 999 times. The standard deviations of observed βMNTD (βMNTD_obs_) and the mean of βMNTD_null_ are expressed as β-nearest taxon index (βNTI). |βNTI| > 2 indicates that deterministic processes play dominant roles in community assembly, and |βNTI|< 2 indicates that stochastic processes govern the community assembly [32,33,34].

## 3. Results

### 3.1. Clinical Symptoms and Histopathological Observation of White-Gill Diseased L. crocea

Compared with healthy *L. crocea* (Figure S2A–C), white-gill diseased fish showed symptoms of whiter body color, bloodless muscles or thin blood, pale gill, and yellow liver (Figure S2F–H). Most infected fish also exhibited symptoms such as floating at the surface, slow swimming and anorexia.

Histopathological observations showed that the gill tissue of white-gill diseased *L. crocea* was disorganized and the gill lamellae were adherent to each other (Figure S2I). The liver in diseased fish revealed marked hepatocellular damage, characterized by cloudy swelling, hydropic degeneration, and vacuolization of hepatocytes, accompanied by signs of nuclear pyknosis (Figure S2J).

### 3.2. Alpha Diversity of Gut Microbiota in White-Gill Diseased L. crocea

**Figure 2:**
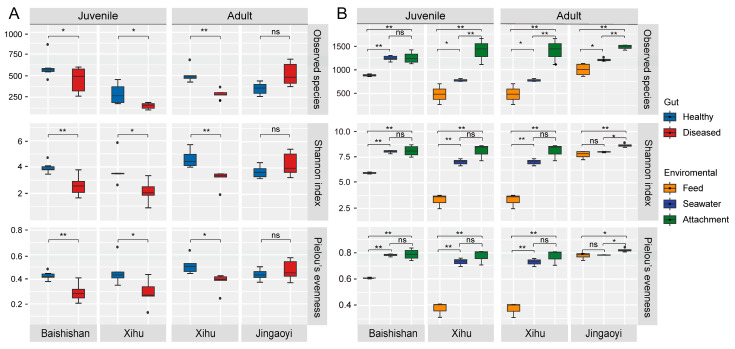
Alpha diversity of bacterial communities in the gut of *Larimichthys crocea* (Richardson, 1846) and the surrounding aquaculture environment. (**A**) Comparison of α-diversity indices (Observed species, Shannon index, and Pielou’s evenness) between healthy and diseased *L. crocea* across different sampling sites and growth stages. Boxes represent the interquartile range, the center line indicates the median, and whiskers denote the minimum and maximum values. Significant differences between the two groups are indicated by asterisks (Student’s *t*-test; * *p* < 0.05; ** *p* < 0.01; ns, not significant). (**B**) α-diversity of bacterial communities in environmental samples, including seawater, feed, and cage attachments. Significant differences among groups are indicated by asterisks (one-way ANOVA with Tukey’s post hoc test; * *p* < 0.05; ** *p* < 0.01; ns, not significant).

The α-diversity of the gut microbiota in *L. crocea*, as measured by Observed species, Shannon index, and Pielou’s evenness, was significantly lower than that of the surrounding environmental microbial communities, including those from seawater, feed, and cage attachments (*p* < 0.05; Figure 2). When comparing healthy and diseased individuals, the gut microbiota of healthy fish (both juveniles and adults) showed significantly higher α-diversity than that of diseased fish across all sampling locations (*p* < 0.05), with the exception of Jingaoyi, where no significant differences were observed.

### 3.3. Compositional Variations in the Gut Microbiota Between Healthy and White-Gill Diseased L. crocea

**Figure 3:**
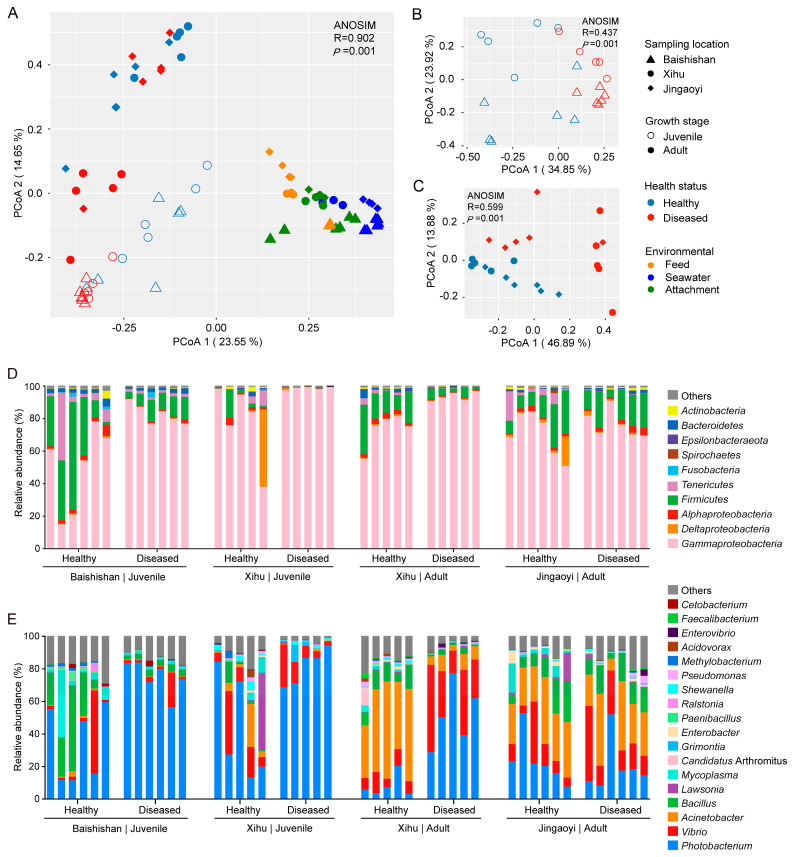
Bacterial community compositions in the guts of healthy and diseased *L. crocea*. (**A**) Principal coordinate analysis (PCoA) based on Bray–Curtis dissimilarity illustrating the compositional variation in bacterial community in the guts of healthy and diseased *L. crocea* and culture environments from different sampling locations. (**B**,**C**) Compositional variations in gut bacterial communities between healthy and diseased juvenile (**B**) or adult (**C**) fish. Analysis of Similarity (ANOSIM) was used to test the significance of the difference between groups. (**D**,**E**) Compositions of the dominant bacterial phyla/proteobacterial classes (**D**) and genera (**E**) (average relative abundance > 3%) in the gut of healthy and diseased fish.

In the PCoA plot based on Bray–Curtis dissimilarity, gut microbiota samples from *L. crocea* were clustered separately from environmental microbiota samples, indicating distinct microbial community compositions between the host-associated and environmental niches (Figure 3A). Within the gut microbiota of *L. crocea*, clear compositional differences were observed based on growth stage (juvenile vs. adult), sampling location (Baishishan, Xihu, and Jingaoyi), and health status (healthy vs. white-gill diseased). PERMANOVA analysis further confirmed that growth stage (28.83%, *p* < 0.001) was the primary factor shaping gut microbial community structure in *L. crocea*, followed by sampling location (26.58%, *p* < 0.001) and health status (11.30%, *p* < 0.001; Table S2).

Given the strong influence of growth stage on gut microbiota composition, juvenile and adult groups were further analyzed separately. In both juveniles (Figure 3B) and adults (Figure 3C), a clear compositional separation was observed between healthy and diseased fish. This distinction was further supported by PERMANOVA, which identified health status as the main contributor to gut microbial variation in juveniles (23.33%, *p* < 0.001) and adults (22.01%, *p* < 0.001; Table S3). Sampling location also had a notable impact, particularly in juveniles (17.38%, *p* < 0.001), whereas its effect was less pronounced in adults (6.62%, *p* < 0.001; Table S3).

Based on sampling location and growth stage, all gut samples were categorized into four groups (Baishishan Juvenile, Xihu Juvenile, Xihu Adult, and Jingaoyi Adult). Within each group, significant differences in gut microbiota composition were observed between healthy and white-gill diseased individuals (ANOSIM, *p* < 0.01; Figure S3A). Furthermore, in juveniles from Baishishan and Xihu, white-gill diseased individuals exhibited significantly lower Bray–Curtis dissimilarity compared to their healthy counterparts (independent t-test, *p* < 0.01; Figure S3B), indicating a convergence in gut microbial community structure among diseased juvenile *L. crocea*. In contrast, no such pattern was observed in adults.

These findings suggest that both intrinsic factors (e.g., growth stage, health status) and extrinsic factors (e.g., environmental conditions at different sampling locations) jointly shape the gut microbiota of *L. crocea*, with health status (i.e., white-gill disease) playing a critical role in gut microbiota differentiation.

### 3.4. Differential Taxa in the Gut Microbiota Between Healthy and White-Gill Diseased L. crocea

The gut microbiota of *L. crocea* was primarily dominated by members of *Gammaproteobacteria* and *Firmicutes* (Figure 3D). In white-gill diseased individuals, the relative abundance of *Gammaproteobacteria* was markedly elevated compared to healthy fish, whereas the relative abundances of *Firmicutes* and *Alphaproteobacteria* were reduced. At the genus level, although notable compositional differences were observed between juveniles and adults, both groups showed a consistent pattern of increased *Photobacterium* abundance following disease onset (Figure 3E). In particular, *Photobacterium* became the overwhelmingly dominant genus in diseased juvenile fish, with its average relative abundance rising from approximately 38% in healthy individuals to 78% in diseased ones. In adult fish, disease was associated with a significant increase in the relative abundances of *Photobacterium* and *Vibrio*, accompanied by a decline in *Bacillus* and *Acinetobacter*, suggesting a shift in the gut microbial community toward potentially pathogenic taxa.

**Figure 4:**
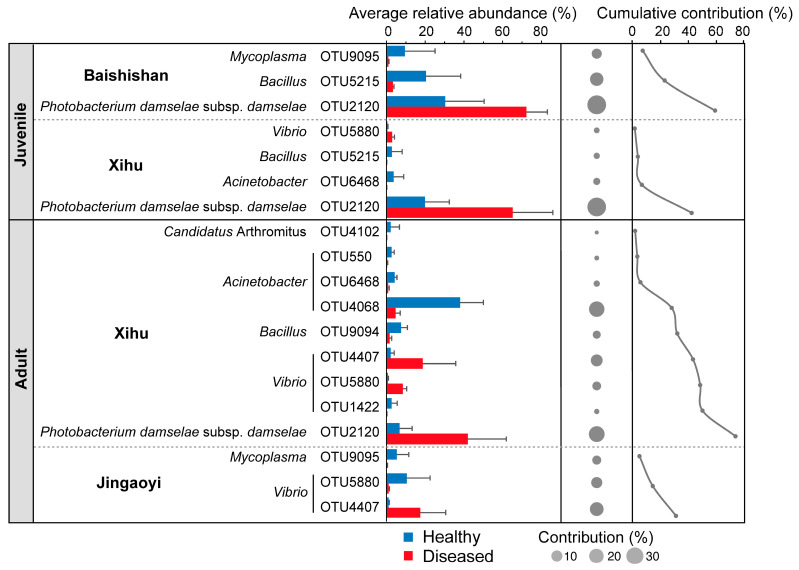
Similarity Percentage (SIMPER) analysis showing the primary contributing OTUs (contribution > 1%) to the dissimilarity of gut bacterial communities between healthy and diseased *L. crocea*. The bars on the left indicate the average relative abundance of each OTU in the healthy or diseased group. The middle and right panels show the dissimilarity contribution percentage and cumulative contribution percentage of OTUs, respectively.

SIMPER analysis was conducted to further identify the key taxa contributing to the dissimilarity in gut microbial communities between healthy and diseased *L. crocea* (Figure 4). The results revealed that *Photobacterium damselae* subsp. *damselae* (OTU2120) was the primary contributor to the observed differences, with its relative abundance reaching up to 90% in diseased fish. In addition, a marked increase in *Vibrio* (OTU4407) was observed in diseased adult fish. Conversely, several taxa were more abundant in healthy individuals, including members of the genera *Bacillus* (OTU5215, OTU9094) and *Acinetobacter* (OTU6468, OTU4068, OTU550), whose relative abundances were significantly higher than in diseased fish. These findings suggest that specific opportunistic pathogens are enriched in diseased hosts, while beneficial or commensal taxa are depleted, highlighting microbial signatures potentially associated with disease progression.

### 3.5. Co-Occurrence Network of Gut Microbiota in White-Gill Diseased L. crocea

**Figure 5:**
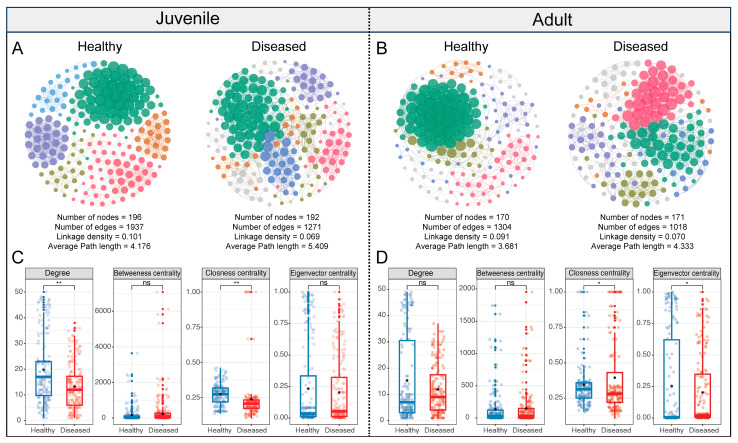
Co-occurrence networks of gut bacterial community in healthy and diseased *L. crocea*. (**A**,**B**) Network visualization comparing co-occurrence relationships of gut bacterial communities between healthy and diseased juvenile (**A**) or adult (**B**) fish. Nodes indicate individual OTUs, while edges represent a significant correlation between OTUs. The size of each node is proportional to its degree. The colors of nodes indicate different network modules. (**C**,**D**) Comparison of network topological features of bacterial communities between healthy and diseased juvenile (**C**) or adult (**D**) fish. Significant differences among groups are indicated by asterisks (Independent Samples T-Test; * *p* < 0.05; ** *p* < 0.01; ns, not significant).

Co-occurrence networks of gut microbiota were constructed for both healthy and diseased fish at juvenile (Figure 5A) and adult stages (Figure 5B). Analysis of network topological parameters revealed that the gut microbial network of juveniles had a greater number of nodes and edges compared to that of adults (Figure 5C,D). In both juvenile and adult groups, the gut microbial networks of diseased fish showed reduced complexity and connectivity compared to those of healthy fish. Specifically, diseased individuals exhibited decreases in the number of edges, linkage density, node degree, and eigenvector centrality, along with an increase in average path length (Figure 5C,D).

### 3.6. Assembly of Gut Microbiota in White-Gill Diseased L. crocea

**Figure 6:**
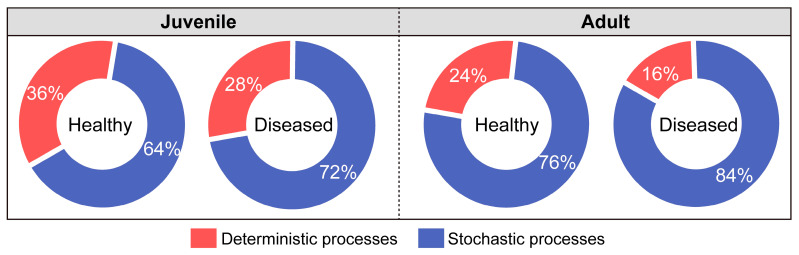
Comparison of ecological processes governing gut bacterial community assembly in healthy and diseased *L. crocea*.

Overall, stochastic processes played a dominant role (64–84%) in the gut microbial community assembly of *L. crocea* (Figure 6). The contribution of stochasticity was generally higher in adults (average 80%) than in juveniles (average 68%). In both growth stages, white-gill disease was associated with increased stochasticity and reduced determinism in community assembly. Specifically, in juveniles, the relative contribution of stochastic processes increased from 64% in healthy individuals to 72% in diseased ones, while in adults, it rose from 76% to 84%.

## 4. Discussion

### 4.1. Reduced Diversity and Stability of the Gut Microbiota in White-Gill Diseased L. crocea

The gut microbiota, often referred to as the host’s “second genome”, plays a crucial role in maintaining host development and health [35]. A diverse gut microbiota is essential for sustaining intestinal homeostasis, enhancing nutrient assimilation, regulating immune responses, and providing colonization resistance against opportunistic microorganisms [36,37]. In the present study, we observed a significant reduction in α-diversity indices (including Observed species, Shannon index, and Pielou’s evenness) in the gut microbiota of white-gill diseased *L. crocea* compared to healthy individuals. This observation is consistent with previous findings in other aquatic species. For instance, Wang et al. [38] reported that *Aeromonas salmonicida*-infected Atlantic salmon (*Salmo salar* L.) displayed significantly reduced gut microbial diversity compared to healthy fish. Similar reductions have also been observed in *Cyprinus carpio* L. with intestinal damage [39] and in *Oncorhynchus mykiss* following bacterial infection [40]. According to the diversity resistance hypothesis, a more diverse microbial community is more likely to be highly competitive and thus more resistant to invasion [41]. In contrast, reduced microbial diversity may create ecological voids or unoccupied niches, facilitating the proliferation of pathogens and increasing the risk of disease outbreaks [42]. Thus, the reduced diversity observed in the gut microbiota of diseased *L. crocea* may compromise the protective capacity of the microbial community and predispose the host to infection or exacerbate disease severity.

Although an overall trend of reduced gut microbial diversity was observed in white-gill diseased fish, minor site-specific deviations were also noted. For instance, diseased fish from Jingaoyi showed a slightly higher mean α-diversity than their healthy counterparts, although this difference was not statistically significant. Notably, fish from Jingaoyi experienced the longest rearing duration among all sites, suggesting that host developmental stage and prolonged culture period may partially account for this localized variation in diversity patterns. Such age-related modulation of the gut microbiota has been widely recognized in fish and may buffer or obscure disease-associated diversity shifts under specific conditions [43,44]. Importantly, these site-specific nuances do not weaken our central conclusion. The predominant pattern of significantly reduced gut microbial diversity in white-gill diseased fish remained consistent and robust across most sampling locations and developmental stages, underscoring dysbiosis as a core microbial feature of the disease.

Beyond diversity metrics, the ecological stability of the gut microbiota may also be compromised in diseased individuals. The microbial community in the gut forms a complex interaction network, where stability is closely tied to the richness and interconnectivity of microbial taxa [45,46]. Generally, higher microbial diversity supports more complex interactions, which in turn contribute to a stable and resilient gut ecosystem [47]. In line with previous reports on aquaculture species such as *Litopenaeus vannamei* [48] and *Plecoglossus altivelis* [49], our co-occurrence network analysis revealed substantial simplification of microbial networks in white-gill diseased fish. Compared to healthy individuals, diseased fish exhibited networks with fewer nodes and edges, reduced linkage density and eigenvector centrality, and increased average path length. These topological changes suggest a fragmentation of microbial relationships and a loss of keystone taxa that normally maintain community cohesion. While it is challenging to definitively establish causality from our data, the observed network simplification is consistent with being both a manifestation of the diseased state and a contributing factor to its progression. The disease likely disrupts the habitat filter and energetic constraints that normally structure the network, leading to its disintegration. This breakdown, in turn, reduces functional redundancy and ecological resilience, potentially creating opportunities for opportunistic pathogens to establish and exacerbate the dysbiosis [50].

### 4.2. Microbial Imbalance Driven by P. damselae Proliferation and Bacillus Reduction in the Gut of White-Gill Diseased L. crocea

In addition to reductions in microbial diversity and network stability, white-gill diseased *L. crocea* exhibited pronounced shifts in gut microbial composition, most notably the significant enrichment of *P. damselae* subsp. *damselae* and concomitant depletion of beneficial genera such as *Bacillus*. These opposing trends highlight a disrupted ecological balance that may play an important role in disease progression.

*Bacillus* spp. (e.g., *B. subtilis* and *B. licheniformis*) are widely regarded as beneficial gut microbes in fish [51,52]. These bacteria are known to produce antimicrobial peptides, antibiotics, and digestive enzymes, which can enhance host immune function, support nutrient metabolism, and inhibit colonization by pathogenic microbes [53]. In our study, the relative abundance of *Bacillus* was significantly higher in the healthy group, suggesting a potential role in maintaining microbial balance and gut health. The decline of such beneficial taxa in diseased fish may weaken microbial-mediated defense mechanisms, allowing opportunistic pathogens to exploit newly available niches in the disturbed intestinal environment [54].

In contrast, *P. damselae* subsp. *damselae* was significantly enriched in diseased fish, becoming the overwhelmingly dominant genus in the gut microbiota, especially in juveniles. SIMPER analysis confirmed that this taxon was the top contributor to microbial dissimilarity between health states, highlighting its potential importance in disease progression. *P. damselae* is a globally distributed marine pathogen consisting of two subspecies: *P. damselae* subsp. *piscicida*, which is host-specific to fish, and *P. damselae* subsp. *damselae*, which exhibits broader host ranges and can infect fish, crustaceans, and mollusks [55,56]. There are significant differences in the clinical manifestations between white-gill disease and those caused by *P. damselae* subsp. *damselae* infection. Clinically, white-gill disease is primarily characterized by pallor and whitening of the gills, respiratory distress, lethargy, and progressive hypoxia, usually without severe hemorrhagic lesions or systemic septicemia [5]. In contrast, infections caused by *P. damselae* subsp. *damselae* are typically associated with acute hemorrhagic manifestations, including extensive bleeding in the gills, fins, and abdominal cavity, tissue necrosis, ulceration, and rapid mortality, reflecting its strong hemolytic and proteolytic activities [57,58]. These differences indicate that *P. damselae* alone cannot fully explain the core clinical phenotype of white-gill disease, although it may significantly aggravate disease severity under certain conditions.

We propose that *P. damselae* in this context acts not as a sole primary etiological agent causing classic septicemia, but as a decisive opportunistic pathogen within a dysbiotic pathobiome. The gut ecosystem in these fish was preconditioned for collapse, as evidenced by the significant loss of diversity and beneficial taxa. This dysbiosis likely impaired the host’s resistance, creating an opportunity for *P. damselae* to proliferate. Its expansion to dominance then became a key driver of pathology, potentially through mechanisms that differ from, or are a subset of, its full virulent repertoire, leading to the specific white-gill presentation. Thus, while *P. damselae* is undoubtedly a critical player; its role is likely enabled and amplified by the underlying microbial community imbalance.

Environmental factors may further modulate this process. White-gill disease typically outbreaks during summer. The water temperatures recorded in this study (28.2–28.8 °C) fall within a range that previous studies have demonstrated enhances the adhesion, growth, and virulence of *P. damselae* subsp. *damselae* on fish mucosal surfaces [59]. At the same time, thermal stress may impair the persistence of probiotic genera such as *Bacillus* by reducing their competitive fitness, antimicrobial production, and colonization resistance, thereby weakening the ecological barriers that normally constrain opportunistic pathogens [60]. *P. damselae* subsp. *damselae* may originate from both environmental reservoirs and resident gut populations. Increased environmental abundance during warm seasons may facilitate its colonization probability, while relaxation of host selective pressure during stress or disease may allow its proliferation from a subdominant state. Although the present study cannot definitively distinguish between these sources, the consistent enrichment of *P. damselae* subsp. *damselae* in diseased individuals support their role as a key ecological indicator associated with dysbiosis and disease progression.

It should be noted that the present analyses are based on relative abundance data derived from 16S rRNA sequencing, which inherently reflect compositional structure rather than absolute microbial load. Future studies incorporating quantitative approaches such as qPCR or spike-in normalization would be valuable to determine whether the observed enrichment of *P. damselae* subsp. *damselae* corresponds to true biomass expansion or primarily reflects proportional restructuring of the community.

### 4.3. Enhanced Stochasticity in Gut Microbiota Assembly of White-Gill Diseased L. crocea

Understanding the ecological processes underlying gut microbiota assembly is essential for identifying how host-associated microbial communities respond to development and disease. Community assembly is controlled by both deterministic processes and stochastic processes. Deterministic processes include host selection, environmental filtering, and interspecific microbial interactions, while stochastic processes are governed by random events such as birth, death, immigration, and extinction [32,61,62]. It is now widely accepted that both processes act simultaneously in community assembly, and the relative contribution of each can vary depending on host condition, environmental context, and disease status [63,64].

In this study, the gut microbiota of *L. crocea* was significantly distinct from that of the surrounding environmental samples, including seawater, feed, and cage surface attachments, regardless of the fish’s health status. This observation reveals the influence of host-driven selection (i.e., deterministic processes) in shaping the gut microbial community, resulting in a compositionally distinct and relatively stable microbiota compared to that of the external environment [65]. While environmental microorganisms act as a microbial source pool, the final structure of the gut microbiota is primarily governed by host-associated factors such as immune regulation and intestinal physiology [66,67]. Quantitative analysis of community assembly further revealed that deterministic processes contributed more to gut microbiota assembly in juvenile fish (average 32%) than in adults (20%). This may be attributed to the active development of the digestive and immune systems in juveniles, which likely imposes stronger selective pressures on microbial colonization, thereby promoting more deterministic assembly patterns. As fish mature, decreased immune plasticity and increased environmental exposure may reduce host selecting capacity and shift the balance of assembly processes toward greater stochasticity [68].

Indeed, our analysis showed that stochastic processes overwhelmingly dominated the gut microbiota assembly in *L. crocea* and became even more dominant in adult fish. More importantly, the contribution of stochasticity was significantly higher in white-gill diseased individuals across both juvenile and adult groups. This shift suggests that disease disrupts host-mediated environmental filtering and selective pressures, thereby weakening deterministic control and allowing for more random colonization and ecological drift within the gut microbiome. This finding is consistent with similar studies in shrimp and fish species, where infection-induced inflammation or immune suppression weakened host control and promoted stochasticity in microbiota assembly [16,69,70]. This shift toward stochastic processes under disease conditions may allow opportunistic pathogens to exploit the loss of probiotic colonization niches [63]. In the case of white-gill disease in *L. crocea*, elevated stochasticity may increase host susceptibility to pathogenic bacteria or heighten the risk of secondary infection, both of which may aggravate disease severity and compromise host health.

Taken together, our results support a bidirectional feedback loop rather than a simple unidirectional causality. Under healthy conditions, beneficial taxa such as *Bacillus* contribute to gut homeostasis by maintaining microbial balance and suppressing pathogen overgrowth. Their depletion, together with environmental stressors, may predispose the host to microbial instability and increase disease susceptibility. Once disease is established, host physiological disruption further weakens deterministic microbial selection, facilitating the expansion of opportunistic taxa such as *P. damselae* and the breakdown of cooperative microbial networks, thereby accelerating the progression of dysbiosis. Therefore, gut microbiota dysbiosis in white-gill diseased *L. crocea* likely functions both as a contributing factor and a consequence of disease development, forming a bidirectional feedback loop. While the present study provides correlative evidence for this interaction, future longitudinal sampling, controlled infection studies, or modeling approaches will be necessary to further disentangle temporal causality and validate specific mechanistic pathways.

## 5. Conclusions

Although the precise etiological agent of white-gill disease requires further elucidation, our study demonstrates that gut microbiota dysbiosis is a central component of its pathogenesis. We identified a consistent dysbiotic signature in diseased *L. crocea*, characterized by reduced microbial diversity, simplified co-occurrence networks, and a marked enrichment of the opportunistic pathogen *P. damselae* subsp. *damselae*, and a decline in beneficial taxa such as *Bacillus*. Furthermore, our analysis of community assembly revealed a significant increase in stochastic processes in diseased fish, indicating a breakdown of host-mediated selective pressures. This finding, integrated with the specific taxonomic shifts, leads us to propose a bidirectional feedback loop as a key model for the disease dynamics: an initial dysbiosis may increase host susceptibility, and the ensuing disease state, by weakening host control, further amplifies the microbial imbalance, creating a vicious cycle that drives disease progression. Collectively, our findings provide a microecological framework for understanding white-gill disease. This new perspective shifts the focus from a singular pathogen to the stability of the host-microbe ecosystem, thereby establishing a scientific foundation for developing novel, microbiome-based strategies for disease prevention and control in *L. crocea* aquaculture.

## Data Availability

The sequence data presented in this study are openly available in [DDBJ] at [https://ddbj.nig.ac.jp/search, accessed on 26 November 2025, accession number: DRA021213]. Further inquiries can be directed to the corresponding authors.

## Supplementary Materials

The following supporting information can be downloaded at: https://www.mdpi.com/article/10.3390/microorganisms13122737/s1, Figure S1: Rarefaction curves showing *α*-diversity indices (including observed species, Shannon index, and Pielou’s evenness) of each sample at different sequencing depths; Figure S2: Anatomical observation and histopathological examination of healthy and white-gill diseased *Larimichthys crocea* (Richardson, 1846); Figure S3: Dissimilarity in gut bacterial community structure between health and diseased *L. crocea* from different sampling locations; Table S1: Detailed information on the environmental parameters of each sampling site; Table S2: Permutational multivariate analysis of variance (PERMANOVA) based on Bray–Curtis dissimilarity with 999 permutations to quantify the effects of different factors on the compositional variation in gut bacterial community of *L. crocea*; Table S3: PERMANOVA based on Bray–Curtis dissimilarity with 999 permutations to quantify the effects of different factors on the compositional variations in gut bacterial communities of juvenile and adult *L. crocea*.
