# Supplementary material for: Microbial Imbalance and Stochastic Assembly Drive Gut Dysbiosis in White-Gill Diseased Larimichthys crocea (Richardson, 1846)
Source: Microorganisms. 2025 Nov 30;13(12):2737. doi: 10.3390/microorganisms13122737 (PMC12735229; doi:10.3390/microorganisms13122737)
Supplement: Supplementary file 1 [file microorganisms-13-02737-s001.zip › microorganisms-3921783-supplementary.pdf]

Supplementary Material

Microbial imbalance and stochastic assembly drive gut dysbiosis in white-gill diseased *Larimichthys crocea* (Richardson, 1846)

Xuan Wang [1], Huangwei Cheng [1], Ting Liu [1], Xuelei Wang [2], Xiongfei Wu [2,3], Junqi Yu [1,4], Demin Zhang [1], Weiliang Shen [2,*] and Dandi Hou [1,3,5,*]

[1] State Key Laboratory for Quality and Safety of Agro-Products, School of Marine Sciences, Ningbo University, Ningbo, 315211, China; 2211130115@nbu.edu.cn (X.W.); cheng_huangwei@163.com (H.C.); liuting991122@163.com (T.L.); 13968836902@126.com (J.Y.); zhangdemin@nbu.edu.cn (D.Z.)

[2] Zhejiang Key Laboratory of Aquatic Germplasm Resources, Ningbo Academy of Oceanology and Fishery, Ningbo, 315832, China; xlwang126@163.com (X.W.); wxiongfei@hotmail.com (X.W.)

[3] State Key Laboratory of Mariculture Breeding, Xiamen University, Xiamen, 361000, China

[4] Zhejiang Key Laboratory of Coastal Biological Germplasm Resources Conservation and Utilization, Zhejiang Mariculture Research Institute, Wenzhou, 325005, China

[5] Institute of One Health Science, Ningbo University, Ningbo, 315211, China

[#] These authors contributed equally to this work.

[*] **Corresponding authors**

E-mail addresses: sweleon@163.com (W.S.), houdandi@nbu.edu.cn (D.H.)

Supplementary Figures

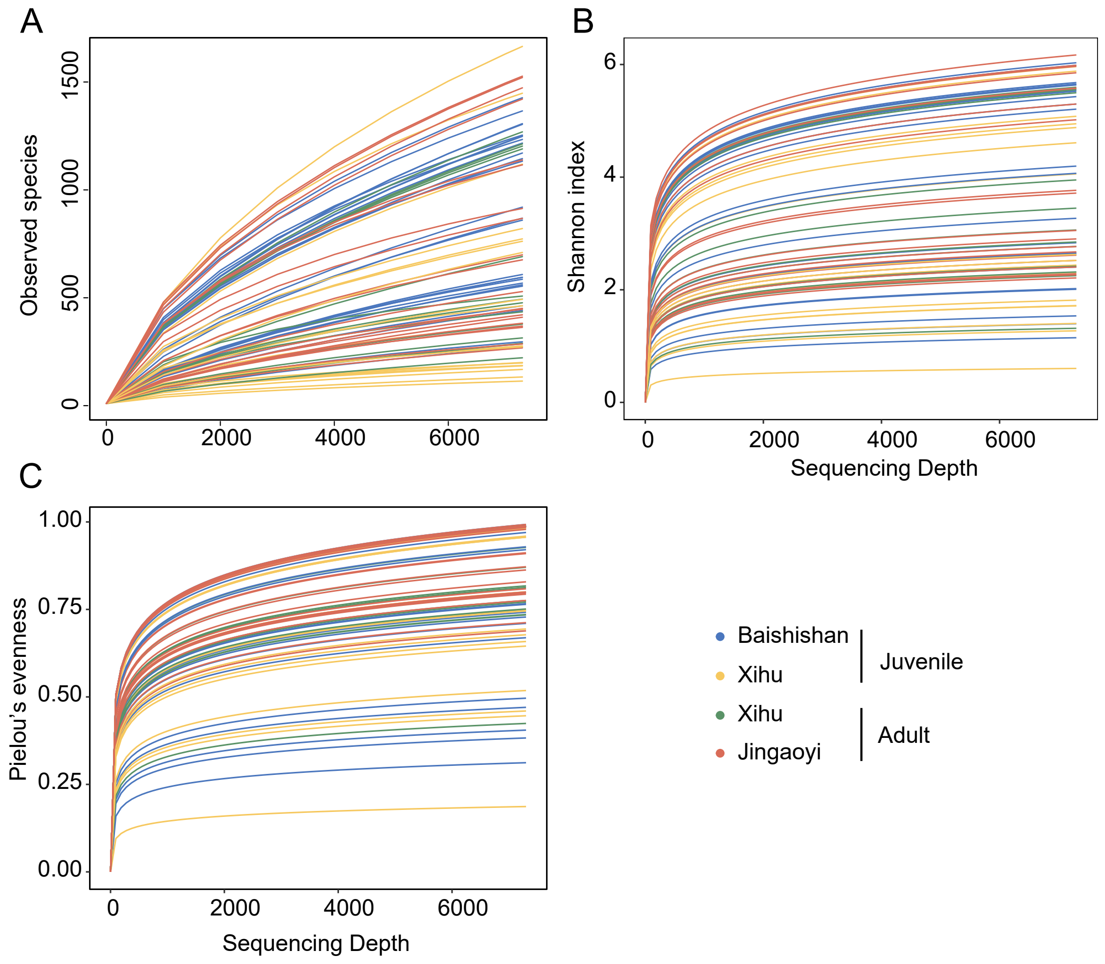

Figure S1. Rarefaction curves showing α-diversity indices (including observed species, Shannon index, and Pielou's evenness) of each sample at different sequencing depths.

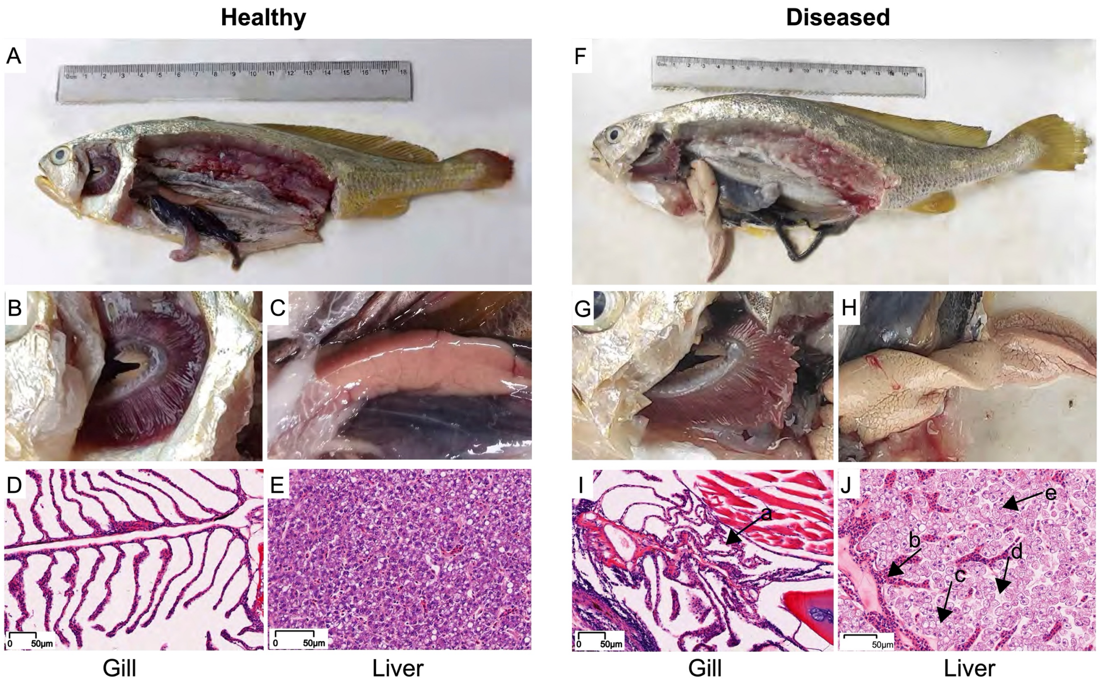

Figure S2. Anatomical observation and histopathological examination of healthy and white-gill diseased *Larimichthys crocea* (Richardson, 1846). Compared with healthy fish **(A-E)**, diseased fish showed symptoms of whiter body color, bloodless muscles or thin blood **(F)**, pale gill **(G)**, and yellow liver **(H)**. The gill **(I)** and liver **(J)** of diseased fish exhibited obvious histopathological features, including (a) disorganized and adherent gill filaments; (b) sinusoidal congestion; (c) hepatocyte vacuolization; (d) sinusoidal dilation; (e) nuclear pyknosis.

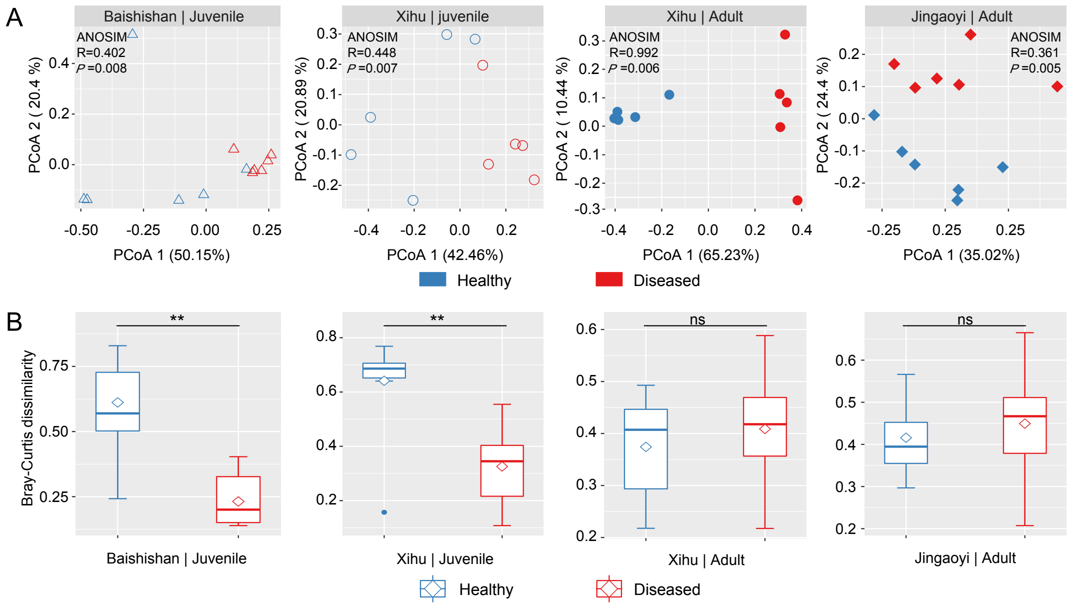

Figure S3. Dissimilarity in gut bacterial community structure between health and diseased *L. crocea* from different sampling locations. **A** Principal coordinate analysis (PCoA) based on Bray-Curtis dissimilarity illustrating compositional variations in gut bacterial community between healthy and diseased fish. Analysis of Similarity (ANOSIM) were used to test the significance of difference between two groups. **B** Comparison of Bray-Curtis dissimilarity of gut bacterial communities within groups of healthy and diseased fish. Significant differences among groups are indicated by asterisks (Independent Samples T-Test; ** $p < 0.01$; ns, not significant).

Supplementary Tables

Table S1. Detailed information on the environmental parameters of each sampling site.

Sampling Site	Sampling Date	Water Temperature (°C)	Salinity	pH	Dissolved Oxygen (mg/L)
**Baishishan**	July 20, 2020	28.6	22.0	7.69	6.63
**Xihu**	August 29, 2020	28.2	21.6	7.52	6.87
**Jingaoyi**	August 15, 2021	28.8	21.8	7.60	5.82

Table S2. Permutational multivariate analysis of variance (PERMANOVA) based on Bray–Curtis dissimilarity with 999 permutations to quantify the effects of different factors on the compositional variation in gut bacterial community of *L. crocea.*

Factor	% explained	R^2	*P*
**Growth stage**	28.83	0.288	0.001
**Sampling location**	26.58	0.266	0.001
**Health status**	11.30	0.113	0.001

Table S3. PERMANOVA based on Bray–Curtis dissimilarity with 999 permutations to quantify the effects of different factors on the compositional variations in gut bacterial communities of juvenile and adult *L. crocea*.

Growth stage	Factor	% explain	R^2	*P*
**Juvenile**	Health status	23.33	0.233	0.001
	Sampling location	17.38	0.174	0.001
**Adult**	Health status	22.01	0.22	0.001
	Sampling location	6.62	0.066	0.001
